# Treatment of cotton with plant growth‐promoting rhizobacteria consortium alters host location and oviposition of *Spodoptera exigua*

**DOI:** 10.1002/ps.70789

**Published:** 2026-04-03

**Authors:** Pascal Mahukpe Ayelo, Basu D. Kafle, Caixing Xiong, Henry Y. Fadamiro

**Affiliations:** ^1^ Department of Entomology Texas A&M University College Station TX USA

**Keywords:** beet armyworm, *Gossypium hirsutum*, integrated pest management, PGPR blends, PGPR‐induced volatiles, avoidance behavior

## Abstract

**BACKGROUND:**

Plant growth‐promoting rhizobacteria (PGPR) trigger induced systemic resistance (ISR) in plants, which could impair herbivore's host location, oviposition behavior and development. A PGPR consortium (*i.e.*, blend of strains) is often more effective than single strains, but its efficacy depends on crop cultivar, herbivore‐crop system, herbivore's degree of specialization, *etc.* In this study, the efficacy of five PGPR consortia, hereafter named TX1, TX2a, TX3, AU8 and AU9a, was evaluated to screen the most promising consortia in controlling the polyphagous insect pest, *Spodoptera exigua* in two cotton cultivars (one susceptible and one resistant to pests). The effects of the PGPR treatments on oviposition behavior, larval development and olfactory responses of *S. exigua*, and on cotton volatile emissions were evaluated.

**RESULTS:**

*Spodoptera exigua* laid significantly fewer eggs on susceptible plants treated with AU8, AU9a, TX1 or TX2a, and on resistant plants treated with AU8, TX1 or TX3, compared to untreated plants. However, the difference in larval survival rates (81–83% in untreated plants *versus* 66–71% in PGPR‐treated plants) was not significant. Y‐tube olfactometer bioassays using the promising PGPR treatments revealed that headspace volatiles of susceptible plants treated with AU8 or TX1, and resistant plants treated with AU8, TX1 or TX3 were avoided by *S. exigua* when compared against blank air, indicating an inhibitory effect on *S. exigua* behavior. Gas chromatography‐mass spectrometer analysis of headspace volatiles revealed that the most discriminant compounds between PGPR‐treated and untreated plants were *α*‐pinene, *β*‐pinene, benzaldehyde, D‐limonene, (*Z*)‐3‐hexenyl acetate, *β*‐ocimene and DMNT. These compounds are known to modulate herbivore's host location and oviposition behavior.

**CONCLUSION:**

This study identifies two promising PGPR consortia (AU8 and TX1) and potential repellent compounds for the protection of cotton cultivars against *S. exigua* and discusses their role as part of an integrated pest management (IPM) approach. © 2026 The Author(s). *Pest Management Science* published by John Wiley & Sons Ltd on behalf of Society of Chemical Industry.

## Graphical abstract

Two plant‐growth promoting rhizobacteria (PGPR) consortia (AU8 and TX1) induced a deterrence of host plant location and oviposition behavior of *Spodoptera exigua* females in both susceptible and resistant cotton plants. Most of the volatile organic compounds (VOCs) increased in PGPR‐treated plants compared to untreated plants. # indicates treatments not selected for olfactory bioassays.

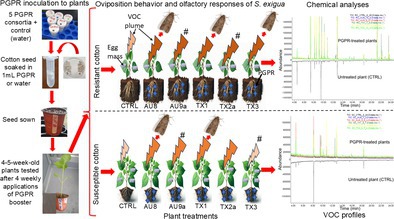

## 1 INTRODUCTION

Cotton (*Gossypium hirsutum* L.) (Malvaceae) is one of the economically important crops grown in many regions worldwide to produce fiber and oil.^1^, ^2^ However, this crop is damaged by a wide range of insect pests (approximately 200 species) that cause approximately 15% of yield losses annually.^1^, ^3^ The beet armyworm, *Spodoptera exigua* (Hübner) (Lepidoptera: Noctuidae) is a highly destructive insect pest and one of the top 10 lepidopteran species that cause economic damage to cotton.^4^ *Spodoptera exigua* is a polyphagous herbivore, feeding on a wide range of hosts, approximately 170 plant species from 35 families.^5^, ^6^ To control this insect pest in cotton, producers typically rely on planting transgenic Bt‐cotton varieties and applying conventional synthetic insecticides,^7^, ^8^ but these control methods are associated with risks of development of resistance in the insect pest and negative impacts on the environment and on human health.^7^, ^8^ Management of the plant natural resistance is a promising alternative for the sustainable control of *S. exigua* through an integrated pest management (IPM) approach.^9^, ^10^, ^11^

Plant growth‐promoting rhizobacteria (PGPR) are beneficial soil bacteria that naturally colonize plant roots and induce multiple mechanisms to boost plant growth and resistance. Application of PGPR to plants has become a supplement or substitute to the use of chemical fertilizers in many crops, playing a crucial role in mobilizing nutrients and improving their uptake by plants to enhance plant growth.^12^, ^13^ Moreover, PGPR are reported to trigger induced systemic resistance (ISR) in plants, leading to changes in the production of allelochemicals and metabolites that impair the host location and oviposition behavior, and development of herbivorous insects, thereby playing a key role in IPM.^14^, ^15^ Several studies have demonstrated that a PGPR consortium (*i.e*., blend of compatible strains) is more effective than single strains in promoting plant growth and inducing systemic resistance against biotic stressors including pathogens and herbivorous insects.^16^, ^17^ However, the efficacy of a PGPR consortium varies, depending on crop cultivar, herbivore‐crop system, degree of specialization and feeding guild of the herbivore, compatibility between strains used in the consortium, *etc*.^18^, ^19^, ^20^ Other challenges which constrain PGPR effectiveness and large‐scale adoption include antagonism relationships between applied PGPR and the indigenous soil microbiomes, including plant natural symbionts,^18^, ^21^ variable environmental conditions, local agricultural practices, soil properties, *etc*.^22^, ^23^ Previous studies focused mostly on testing the effects of PGPR on oviposition behavior and larval development using individual insect species in a single crop cultivar,^15^, ^24^ and there are very few studies comparing the efficacy across multiple PGPR consortia.^25^, ^26^ On the other hand, the olfactory responses of *S. exigua* to plant volatiles, and the identification of volatile compounds that mediate interactions between the insect and its host plants have been overlooked, and only a few studies reported some compounds from rose plant, *Rosa chinensis* Jacq. (Rosaceae) and rosemary plant, *Salvia rosmarinus* Spenn. [formerly *Rosmarinus officinalis* L.] (Lamiaceae).^27^, ^28^, ^29^ However, extensive research in this area has been conducted for other insect‐plant interactions.^14^, ^15^

Evaluation of the effectiveness of PGPR in crop cultivars that are resistant to pathogens and insect pests has received limited attention in previous studies, but this is crucial for the development of PGPR consortia that have broad‐spectrum efficacy. In a recent study, using two corn and two cotton cultivars of contrasting levels of resistance to pests, we tested five PGPR consortia and reported that all consortia promote the growth of root and shoot of the susceptible cultivars (cotton Acala 1517‐18 GLS and corn B73), but some consortia were unable to promote the growth of the resistant cultivars (corn CML 176 and cotton Acala 1517‐08).^30^ The PGPR consortia consisted of three new PGPR formulations, hereafter named TX1, TX2a and TX3 (made of PGPR strains recently collected from the rhizosphere of corn and wheat field crops in Texas, USA) and two previous formulations (AU8 and AU9a made of strains isolated from the rhizosphere of corn, cotton and cucumber in Auburn, Alabama, USA). Here, the effectiveness of the five PGPR consortia in inducing resistance responses in both the susceptible and resistant cotton cultivars for the control of *S. exigua* was evaluated to identify the most promising consortia that could be used as part of an IPM approach in cotton fields. Specific objectives of this study were to (i) evaluate the effects of the PGPR treatments on the host location, oviposition behavior, and development of *S. exigua*, and (ii) identify the PGPR‐induced volatile organic compounds mediating the observed responses consistent with ISR‐mediated effects against *S. exigua*. The findings should inform the development of PGPR consortia that are effective in controlling *S. exigua* across multiple cotton cultivars.

## 2 MATERIALS AND METHODS

### 2.1 Preparation of PGPR consortium suspension

Five PGPR consortia (TX1, TX2a, TX3, AU8 and AU9a) were tested in this study, and their compositions are presented in Table 1. The PGPR consortia were formulated in a way that each consortium is made of strains that do not exhibit antagonism against one another in preliminary bioassays by co‐inoculation of strains on agar plate through antibiosis bioassays that revealed no clear zone of inhibition, as described in a previous study.^31^ While AU9a contains strains from one bacterial taxonomic group (*Bacillus*), the other consortia contain strains from two or three groups: *Bacillus* and *Fictibacillus* for AU8; *Bacillus* and *Paenibacillus* for TX1; *Bacillus, Peribacillus* and *Priestia* for TX2a; and *Bacillus, Paenibacillus* and *Priestia* for TX3. The preparation of the PGPR consortia followed the procedure described in our previous study.^30^ The PGPR strains were equally abundant in each consortium with a concentration of 1 × 10^7^ CFU/mL (colony forming unit per milliliter) for each strain. The consortia AU8, TX1 and TX3 were made of four strains and had a final bacterial concentration of 4 × 10^7^ CFU/mL, while AU9a and TX2a made of three strains had a final bacterial concentration of 3 × 10^7^ CFU/mL. AU9a and TX2a contained, respectively, the same strains in consortia AU9 and TX2 tested in our previous studies,^30^, ^32^ except *Bacillus velezensis* AP‐295 and *Pantoea dispersa* TC33, respectively. These two strains were not tested in this study because *B. velezensis* AP‐295 became unavailable while *P. dispersa* TC33 was recently reported to be potentially harmful to human and plants.^33^

**Table 1:** Identity of the PGPR strains and composition of consortia (blends) used in this study.

Consortia	Strain ID	Organism name^a^	Host plant^b^	References
TX1	TC04	*Bacillus subtilis*	Corn	Ayelo *et al*.^30^
	TC09	*Bacillus albus*	Corn	Ayelo *et al*.^30^
	TC13	*Bacillus halotolerans*	Corn	Ayelo *et al*.^30^
	TC44	*Paenibacillus alvei*	Corn	Ayelo *et al*.^30^
TX2a	TC01	*Bacillus amyloliquefaciens*	Corn	Ayelo *et al*.^30^
	TC05	*Peribacillus simplex*	Corn	Ayelo *et al*.^30^
	TC11	*Priestia megaterium*	Corn	Ayelo *et al*.^30^
TX3	TW58	*Bacillus inaquosorum*	Wheat	Ayelo *et al*.^30^
	TW59	*Paenibacillus illinoisensis*	Wheat	Ayelo *et al*.^30^
	TW60	*Priestia megaterium*	Wheat	Ayelo *et al*.^30^
	TW62	*Priestia aryabhattai*	Wheat	Ayelo *et al*.^30^
AU8	AP‐188,	*Bacillus velezensis*	Cotton	Disi *et al*.^32^
	AP‐209	*Bacillus mojavensis*	Cotton	Disi *et al*.^32^
	AP‐217	*Fictibacillus solisalsi*	Cucumber	Disi *et al*.^32^
	AP‐218	*Bacillus velezensis*	Cucumber	Disi *et al*.^32^
AU9a	AP‐136	*Bacillus velezensis*	Corn	Disi *et al*.^32^
	AP‐188	*Bacillus velezensis*	Cotton	Disi *et al*.^32^
	AP‐219	*Bacillus velezensis*	Cucumber	Disi *et al*.^32^

^a^ All strains have more than 99% match, and their sequences were deposited in NCBI GenBank: (http://www.ncbi.nlm.nih.gov).

^b^ Indicatesplant from which the PGPR strain was isolated.

### 2.2 Plant production

Two cotton cultivars that have contrasting levels of resistance to pests were used: the glanded cotton cultivar, Acala 1517*–*08 (without Bt) which is resistant to *Fusarium* wilt caused by the pathogen *Fusarium oxysporum f. sp. vasinfectum race 4*, and arthropod herbivores like the corn earworm *Helicoverpa zea* Boddie, and the glandless cultivar, Acala 1517–18 GLS (without Bt) which is susceptible to *Fusarium* wilt and *H. zea*.^34^ Cotton plants were grown under greenhouse conditions (25 ± 2°C and 55 ± 10% RH) with supplemental LED daylight (VYPR 2P Luminaire, Fluence Bioengineering, TX, USA) under a photoperiod regime of 14:10 h (L:D) as described in our previous study.^30^ The cell suspensions of the PGPR consortia were used to inoculate the cotton seeds and applied as booster to seedlings during the plant production. Briefly, seeds were first surface disinfected by soaking in 2% commercial bleach solution for 3 min and rinsing thereafter 10 times with sterile deionized water, after which, each seed was soaked for 15 min in 1 mL of the cell suspension of one PGPR consortium as in previous studies.^30^, ^35^ Untreated seeds were soaked in deionized water and served as control. Thereafter, seeds were individually sown in pot (10.5 × 7 × 8 cm) on Pro‐Line HFC/20 growing mix made of Canadian sphagnum peat moss (60%), hydrafiber and perlite (Jolly Gardener, Atlanta, GA, USA). One millimeter (1 mL) of a PGPR cell suspension was applied to each plant as a booster, once a week over 4 weeks after seeding. Plants were fertilized once (before the second booster application) by applying 35 mL of 250 ppm solution (NPK 24‐8‐16, Miracle‐Gro®, Marysville, OH, USA) to each plant. Four‐ to 5‐week‐old plants were used in the experiments. Twenty‐four hours before the experiments, plants were watered and transferred onto a light‐shelf under a spectrum LED daylight (Patiowell Plant Growing Lamp, Lowe's, Mooresville, NC, USA) of 200 μE m^−2^ s^−1^ light intensity and a photoperiod regime of 14:10 h (L:D) in a laboratory room (26 ± 1°C and 50 ± 10% RH). This allowed acclimatization to experimental conditions before the experiments.

### 2.3 Insect rearing

The colony of *S. exigua* was initiated with eggs purchased from Benzon Research, Inc. (Carlisle, PA, USA) and reared in a laboratory room at Texas A&M University (College Station, TX, USA) under 25 ± 1 °C, 75 ± 5% RH, and 12:12 h (L:D) photoperiod regime. Conical mesh metal wastebaskets (Universal UNV20008 18 Qt.) with clean white cotton cloth on top (serving as lid) were used as rearing containers in which about 40 pairs of moths were kept. Female moths were allowed to oviposit on the cloth for 48–72 h, after which the cloth with the eggs was collected, then placed in Ziploc bag, where eggs hatch into neonate larvae, about 2 or 3 days after egg laying. Thereafter, batches of the neonate larvae were transferred into plastic cups onto a pinto bean‐based diet.^36^ After 5 or 6 days, developing larvae were transferred individually into multicellular clear plastic rearing trays (32 cells) with opaque ventilated lids (Product no. 9074‐L, Frontier Agricultural Science, DE, USA) and kept on diet until they pupate. Determination of moth sex was conducted at pupal stage using the protocol described in a previous study.^37^ Once emerged, male adults were marked by clearing scales from the dorsal thorax with a fine paintbrush and then painted with a drop of white odorless ink. Newly emerged 1‐ or 2‐day‐old adults were allowed to mate for 48 h. Adults were provided with 10% sucrose water solution on cotton wicks. Mated females of 3‐ to 5‐day‐old were used in the oviposition and olfactometer bioassays.

### 2.4 Effect of PGPR treatment on *Spodoptera exigua* oviposition behavior

#### 2.4.1 No‐choice oviposition bioassays

Oviposition by mated *S. exigua* females on the cotton cultivars was evaluated in mesh cages (38 × 38 × 76 cm tall). Plants treated with either PGPR consortium (AU8, AU9a, TX1, TX2a, or TX3) or left untreated (control), were individually placed inside a single mesh cage, with plant pot wrapped in aluminum foil so that only plant stem and leaves were exposed for oviposition. Experimental cages were spaced 120 cm apart, and the positions of treatments were randomized for each test. Cotton wool soaked with 10% sugar solution was placed inside the cage and served as a feeding source for the insects. A group of four *S. exigua* adult females was released into each cage and kept for 16 h overnight (5 pm to 9 am) in a darkened laboratory room as the insect is nocturnal. Thereafter, the moths were removed from the cage, and the numbers of eggs on each plant and cage were visually counted under a Leica STE stereo microscope (Stereozoom, Microsystems, China) at 10× magnification. Egg numbers were recorded separately (on plant *versus* on cage) per plant treatment. A total of 15 or 16 replicates were conducted and used in the data analysis.

#### 2.4.2 Four‐choice oviposition bioassays

The oviposition preference of *S. exigua* to PGPR‐treated plants *versus* untreated plants was evaluated in a four‐choice experiment. Three promising PGPR consortia (those that reduced oviposition in PGPR‐treated plants relative to untreated plants during the no‐choice bioassays) were selected for this experiment, and included AU8, TX1 and TX2a for the susceptible cultivar, and AU8, TX1 and TX3 for the resistant cultivar. Four plant treatments (the three PGPR consortia and one untreated) were arranged 80 cm apart from each other, inside a mesh cage (115 × 115 × 76 cm) and their positions were randomized for each test. Sixteen *S. exigua* females were released at the center of the experimental cage and allowed to oviposit for 24 h (5 am to 5 pm). The cages were covered with black fabric cloth in a way that allowed ventilation and provided darkness throughout the 24 h experimental period. Here, the experimental period was extended to 24 h because several replicates had no eggs on plants in preliminary choice bioassays set for 16 h. At the end of the experiment, the eggs were counted on each plant as described above in the no‐choice bioassays. Replicates for which no eggs were recorded on any of the four plants were discarded (*n* = 4 for the resistant cultivar and *n* = 3 for the susceptible cultivar) because the insects in these replicates were considered non‐responsive and potentially inactive. Only replicates with eggs on at least one plant (total of 17 replicates) were used in the data analysis.

### 2.5 Effect of PGPR treatment on the development of *Spodoptera exigua* larvae

The effect of the PGPR consortia on the development of *S. exigua* larvae was investigated under laboratory conditions at 25 ± 1 °C, 75 ± 5% relative humidity, and 12:12 h (L:D) photoperiod regime using method previously described by Adesemoye *et al*.^38^ with a slight modification. Larval development was monitored on the six treatments (untreated, AU8‐, AU9a‐, TX1‐, TX2a‐ and TX3‐treated plants). Using a fine paintbrush, eight neonate larvae (1‐day‐old) were placed on the top second and third developed leaves (four larvae per leaf) of each plant. Then, each leaf with the larvae was enclosed in a mesh sleeve organza fabric bag (13 × 18 cm) to restrict the movement of the larvae to the specific leaf. In few replicates where the leaf was entirely consumed before the end of the experiment, the larvae were transferred onto another leaf of the same plant. The bioassays were replicated eight to 10 times, eight larvae per replicate, making a total of 80 to 100 larvae monitored per treatment. The development of the larvae was monitored over 10 consecutive days by recording the number of dead larvae per plant on each day, and the larval weights on the third, fifth, seventh and tenth days.

### 2.6 Olfactory responses of *Spodoptera exigua* to PGPR‐treated plant volatiles

A Y‐tube olfactometer (stem arm: 20 cm long; two side arms: 8.5 cm long; internal diameter (i.d.): 2.4 cm) (Sigma Scientific LLC, FL, USA) was used to examine the behavioral responses of *S. exigua* females to volatiles of untreated or PGPR‐treated cotton plants. Teflon PTFE tubing was used to connect each side arm of the Y‐tube to a 5 L glass jar used as a container of the plant treatment. The method followed a protocol previously described by Ayelo *et al*.^39^ with some modifications. Preliminary bioassays in control tests (blank air *versus* blank air, *n* = 60) revealed that proportion of responsive insects was higher when the Y‐tube was placed vertically (62%) compared to horizontally (35%). Therefore, the Y‐tube was oriented vertically in subsequent bioassays. The three promising PGPR consortia tested per cultivar in the oviposition choice experiments were considered for the olfactometer bioassays. The insects were given a choice between blank air (control) and volatiles of an untreated or PGPR‐treated plant. The following dual choice tests were conducted: (i) control *vs*. untreated plant; (ii) control *vs*. AU8‐treated plant; (iii) control *vs*. TX1‐treated plant, (iv) control *vs*. TX2a‐treated plant were tested for the susceptible cotton; and (v) control *vs*. untreated plant, (vi) control *vs*. AU8‐treated plant, (vii) control *vs*. TX1‐treated plant and (viii) control *vs*. TX3‐treated plant for resistant cotton. The plant pot with the potting soil was wrapped with aluminum foil to minimize contamination of the headspace volatiles from the soil and then placed in a 5 L glass chamber serving as odor container. A total of 60 or 70 *S. exigua* females were tested per choice test with 10 insects tested per day per choice test. A single insect was placed in the release zone (4 cm length, 2.4 cm i.d.) connected to the base of the Y‐tube stem and was given 15 min to make a choice. The insect was considered to have made a choice when it walked 4 cm into either side arm and stayed there for 30 s. Insects that did not make a choice by the end of the observation time were considered unresponsive and therefore excluded during data analysis. Whereas insects that did not move out of the release zone were considered unhealthy and were not counted in the test replicates. After 1 h had elapsed or five insects tested (whichever comes first), the Y‐tube glass was replaced with another clean one and the odor sources were switched between left and right of the Y‐tube side arms. Between choice tests on the same day, the Y‐tubes and jar containers were cleaned with acetone, then air‐dried at room temperature before they were re‐used. At the end of each experimental day, the Y‐tubes and jar containers were cleaned with odorless detergent then rinsed with water followed by acetone. A 4‐Port air delivery system with Venturi vacuum (Model: CADS‐4CPP, Sigma Scientific LLC, Micanopy, FL, USA) was used to push purified air into the side arms at a constant flow rate of 300 mL/min. The air was pulled out from the system at the base of the Y‐tube stem at a rate of 600 mL/min. The experiments were conducted in a darkened room to mimic the condition of the nocturnal behavior of the insect. To enable observation for data collection, illumination was provided using a lamp with red light (18 W, 120 V, 250 Lux, Bluex Bulbs™, China) which was hung 30 cm above the olfactometer Y‐tube. Before the start of each test, the plants were left for 2 h inside the containers and the insects allowed 1 h to acclimatize to the darkened conditions prior to start of the experiments. Preliminary observations during nighttime revealed that after 21:00 local time, females were more likely to release anal/pheromonal secretions and lay eggs in the Y‐tube system. Therefore, experiments were conducted between 17:00 and 20:00 local time to reduce chemical contamination in the test arena. During this observation period, the release of the chemical secretion or egg laying occurred rarely, and when this occurred, the test insect was discarded, and the Y‐tube was immediately cleaned with acetone, then air‐dried at room temperature before testing another insect. A total of 60 or 70 replicate insects were tested per dual choice test over 6 or 7 separate days (10 insects tested per day per choice test).

### 2.7 Collection and analysis of volatile organic compounds

Headspace volatiles organic compounds (VOCs) were trapped onto prepacked 50 mg Super‐Q adsorbents (Alltech Associates, Deerfield, IL, USA) using a dynamic pull system as previously described.^40^ Volatiles were collected from plants treated with one of the PGPR consortia (AU8, AU9a, TX1, TX2a or TX3) and untreated plants. The plant pot with the potting soil was wrapped with aluminum foil prior to volatile collection to minimize water evaporation and volatile contamination from the soil. Thereafter, the plant was placed in a volatile collection chamber (5 L glass jar; Sigma Scientific LLC, Micanopy, FL, USA). Headspace air of potting soil without a plant was also collected to check for miscellaneous impurities and background noise. A 4‐Port air delivery system (Model: CADS‐4PushPull, Sigma Scientific LLC, Micanopy, FL, USA) was used to pull clean and purified (using activated charcoal) air stream at a flow rate of 350 mL/min through the collection chambers at room temperature. The headspace volatiles were collected for 24 h, then eluted with 400 μL of methylene chloride (solvent) (≥99.8% purity, Sigma‐Aldrich). The resulting extracts (400 μL) were stored in a freezer at −40 °C until analysis by gas chromatography – mass spectrometry (GC–MS).

The GC–MS system is comprised of an Agilent 8890 GC coupled with a 5977B MS installed with Inert Plus MSD Turbo EI/CI Bundle and an Autoinjector (Agilent Technologies, Chicago, USA). The MS is equipped with a low bleed non‐polar capillary column (5% phenyl and 95% methylpolysiloxane, 30 cm × 0.25 mm × 0.25 μm film thickness). An aliquot of 1 μL of each headspace volatile extract was injected into the GC–MS and analyzed for 35 min. The GC oven was initially set at 40 °C held for 2 min, then programmed to increase at a rate of 5°C/min to reach a final temperature of 200 °C which was held for 1 min. Injector and detector temperatures were set at 200 °C, and spectra were recorded at 70 eV in electron impact (EI) mode. Identification of compounds was based on retention times, Kovats retention indices (RIs) and mass spectra in comparison with those in the libraries of the National Institute of Standards and Technology (NIST20; Gaithersburg, MD, USA). The RIs were calculated using retention times of n‐alkane (C_8_–C_20_) standards which were run as a mixture in a separate injection using the same temperature program. Confirmation of the identity of the compounds was done by comparison of their mass spectra to those of commercially available synthetic standards: *α*‐Pinene, *β*‐Pinene, Benzaldehyde, *β‐*Myrcene, (*Z*)‐3‐Hexenyl acetate, D‐Limonene, Ocimene mixture, Nonanal, Decanal, *β*‐Caryophyllene, α‐Humulene, Farnesene mixture; all purchased from Sigma Aldrich, USA with a purity above 98%, except Nonanal (95%) and Ocimene (90%). These standards were run in GC–MS using the same program used for analyzing the headspace volatile extracts. For quantification, internal calibration was performed, a method suitable for quantifying relative amounts of volatile compounds.^41^, ^42^ Briefly, synthetic of nonyl acetate (purity ≥95%, Sigma‐Aldrich) was used as internal standard by dissolving 1 μL of 2000 ng/μL nonyl acetate solution into the headspace volatile extract volume (V = 400 μL), corresponding to a concentration of 5 ng/μL nonyl acetate in the sample injected into GC–MS. The concentration (Cc) and the release rate (Rr, doses in ng/plant/h) of each identified compound were then calculated using a formula described in previous studies,^39^, ^43^ as follows:

$$\mathrm{Cc}=\frac{\frac{\mathrm{PAc}}{\text{PAis}}\times \mathrm{Cis}\times 1\;\mathrm{uL}}{V}\;\text{and}\;\mathrm{Rr}=\frac{\mathrm{Cc}\times V}{24}$$

PAc and Cc are respectively peak area and concentration of the identified compound. PAic and Cis are respectively peak area and concentration of the internal standard. Rr is the release rate of the identified compound, and V is the volume of headspace volatile extract.

### 2.8 Statistical analysis

Data were checked for normality using Shapiro–Wilk's test, homogeneity of variance using Bartlett's test, and for overdispersion using the mean *versus* variance method where necessary. Egg count data in all comparisons between the six treatments (*i.e*., untreated, AU8‐, AU9a‐, TX1‐, TX2a‐, and TX3‐treated plants) were analyzed using a generalized linear model (GLM) with negative binomial distribution to account for the overdispersion nature of the data.^44^ Data on larval survival rates were analyzed using a GLM with binomial distribution and logit link function.^44^ Comparisons of larval weights and volatile concentrations were performed using the non‐parametric Kruskal–Wallis test, matching one‐way analysis of variance (ANOVA), followed by a post‐hoc Dunn's test with Bonferroni's model to separate the means when a significant difference was noted (*P* < 0.05).^45^ Multivariate analyses were further performed to compare the volatile profiles of the plant treatments using a sparse partial least squared discriminant analysis (sPLS‐DA) and the clustering heatmap, as previously described in Miano *et al*.^46^ Thereafter, an sPLS‐DA biplot was performed in the mixOmics package to illustrate the correlation between the VOCs and the treatments, and the variable importance in the projection (VIP > 1) associated with the sPLS‐DA was used to select the VOCs that best discriminated PGPR‐treated plants from untreated plants (control).^46^, ^47^ The clustering heatmap was built using the function ‘cim’ in the mixOmics package to visualize quantitative variations in the VOCs across replicates of the plant treatments.^47^, ^48^ Validation of the sPLS‐DA model was done using the function ‘perf’ and the sPLS‐DA parameters (R^2^X, R^2^Y and Q^2^) in the mixOmics package.^48^ Frequencies of choices made by the insects in the Y‐tube olfactometer assays were compared using a Chi‐square (χ^2^) goodness of fit test. All data were analyzed using version 4.3.1. of R statistical software.^49^

## 3 RESULTS

### 3.1 Effect of PGPR treatment on *Spodoptera exigua* oviposition behavior

**Figure 1:**
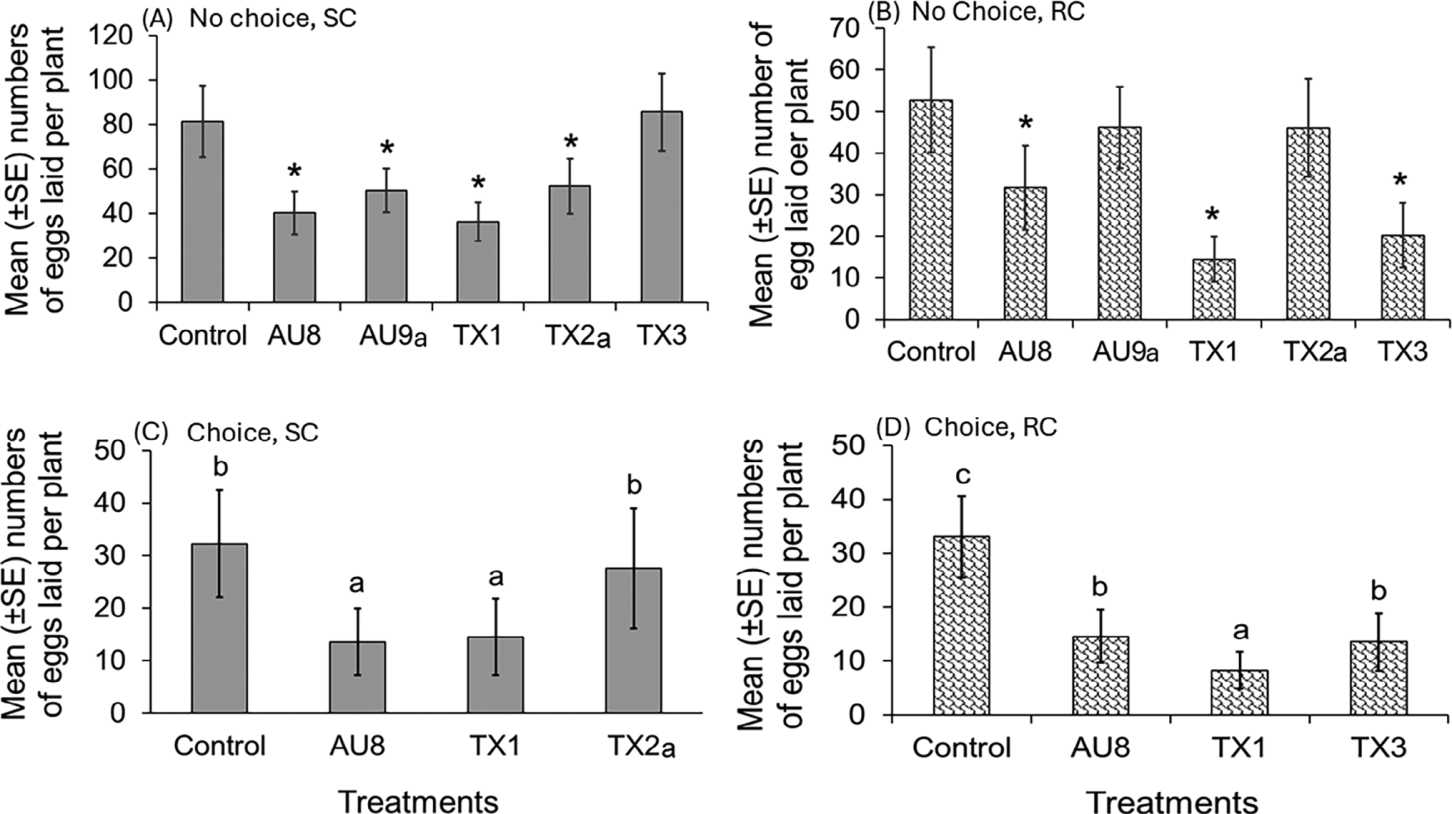
Comparative mean (± SE) numbers of eggs laid by *Spodoptera exigua* between untreated plants (control) and plants treated with the PGPR consortium (AU8, AU9a, TX1, TX2a or TX3). (A) and (B) are respectively no‐choice experiments (15 or 16 replicates) for the susceptible cotton (SC) cultivar (Acala 1517–18 GLS) and resistant cotton (RC) cultivar (Acala 1517‐08), while (C) and (D) are, respectively, the four‐choice experiments using the susceptible and resistant cotton (17 replicates). In the no‐choice experiments, each PGPR treatment is compared to the control, and * indicates significant differences at *P* < 0.05. In the choice experiments, untreated and all PGPR treatments are compared altogether, and different letters indicate significant differences at *P* < 0.05. Data were analyzed using GLM with negative binomial distribution.

Cotton cultivar, PGPR consortium and the interaction of cultivar and PGPR influenced the oviposition behavior of *S. exigua* females (GLM, *P* < 0.001; Fig. 1(A), (B)). In no‐choice experiments, the PGPR consortia AU8 and TX1 induced a significant reduction in egg laying by *S. exigua* on both susceptible and resistant cotton cultivars compared to untreated plants (GLM, *P* < 0.001; Fig. 1(A), (B)). Additionally, *S. exigua* laid significantly fewer eggs on plants of the susceptible cultivar treated with AU9a or TX2a compared to untreated plants (GLM, *P* < 0.001), but no significant differences were recorded in plants of the resistant cultivar (GLM, *P* > 0.05). Whereas the opposite trend was observed for consortium TX3, which induced a significant reduction in egg laying by *S. exigua* on the resistant plants (GLM, *P* < 0.001), but not on the susceptible plants (GLM, *P* = 0.205) compared to untreated plants (Fig. 1(A), (B)). In the experiments where significant differences were noted, the reduction in oviposition due to PGPR treatment was in the range of 35–55% in the susceptible cultivar, and 40 to 72% in the resistant cultivar (Fig. 1(A), (B)).

Results of the four choice bioassays using the most promising PGPR consortia that reduced oviposition by *S. exigua* in the no‐choice experiments indicated that females laid significantly fewer eggs on susceptible plants treated with TX1 or AU8 compared to untreated and TX2a‐treated plants (GML, *P* < 0.001; Fig. 1(C)). For the resistant cultivar, the number of eggs laid by *S. exigua* was the lowest on plants treated with TX1, followed by plants treated with TX3 or AU8, which had significantly fewer eggs relative to untreated plants (GLM, *P* < 0.001; Fig. 1(D)). Here, the oviposition reduction due to PGPR treatment was in the range of 55 to 58% in the susceptible cultivar, and 56 to 75% in the resistant cultivar (Fig. 1(C), (D)).

### 3.2 Effect of PGPR treatment on the development of *Spodoptera exigua* larvae

**Figure 2:**
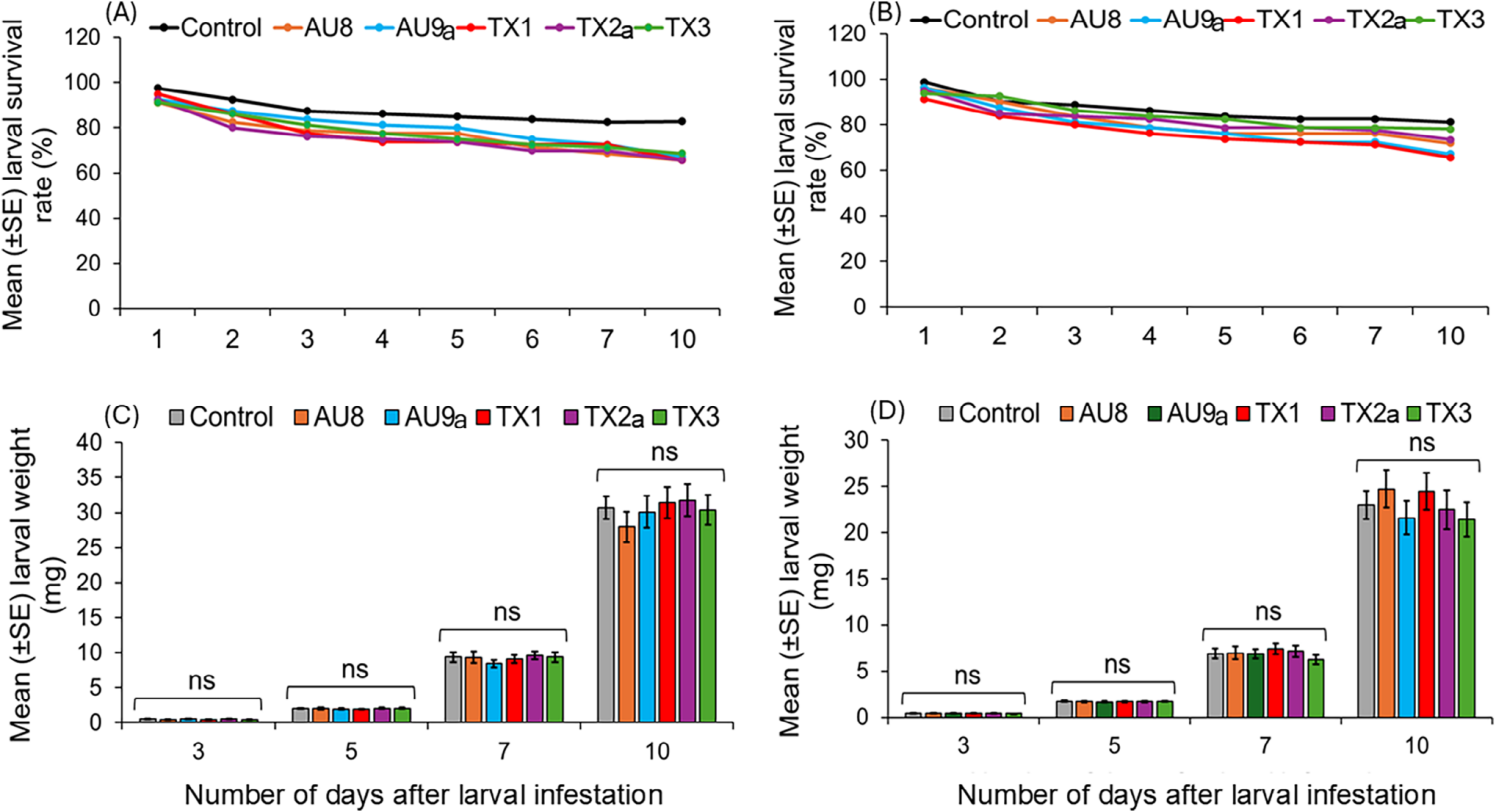
Mean (± SE) survival rate and weight of *Spodoptera exigua* larvae reared on PGPR‐treated plants and untreated plants (control) of the susceptible cotton cultivar, Acala 1517‐18 GLS (A and C) and the resistant cotton cultivar, Acala 1517–08 (B and D). Plants were treated with the PGPR consortium (AU8, AU9a, TX1, TX2a or TX3), or water without any PGPR (control). ns indicates no‐significant differences (GLM, *P* > 0.05).

In the susceptible cultivar, inoculation of the PGPR consortia resulted in 66 to 69% larval survival rate compared to 83% in untreated plants on day 10 post‐release of larvae on plants (Fig. 2(A)). In the resistant cultivar, larvae fed on plants treated with the consortia AU8, AU9a and TX1 exhibited 66 to 71% survival rate compared to 81% in untreated plants (Fig. 2(B)). However, these differences in larval survival rates between PGPR‐treated and untreated plants were not significant in both cultivars (GLM, *P* > 0.05). Similarly, there were no significant differences in the larval weights between untreated and PGPR‐treated plants, both in susceptible and resistant cotton cultivars (GLM, *P* > 0.05; Fig. 2(C), (D)).

### 3.3 Olfactory response of *Spodoptera exigua* to PGPR‐induced cotton volatiles

**Figure 3:**
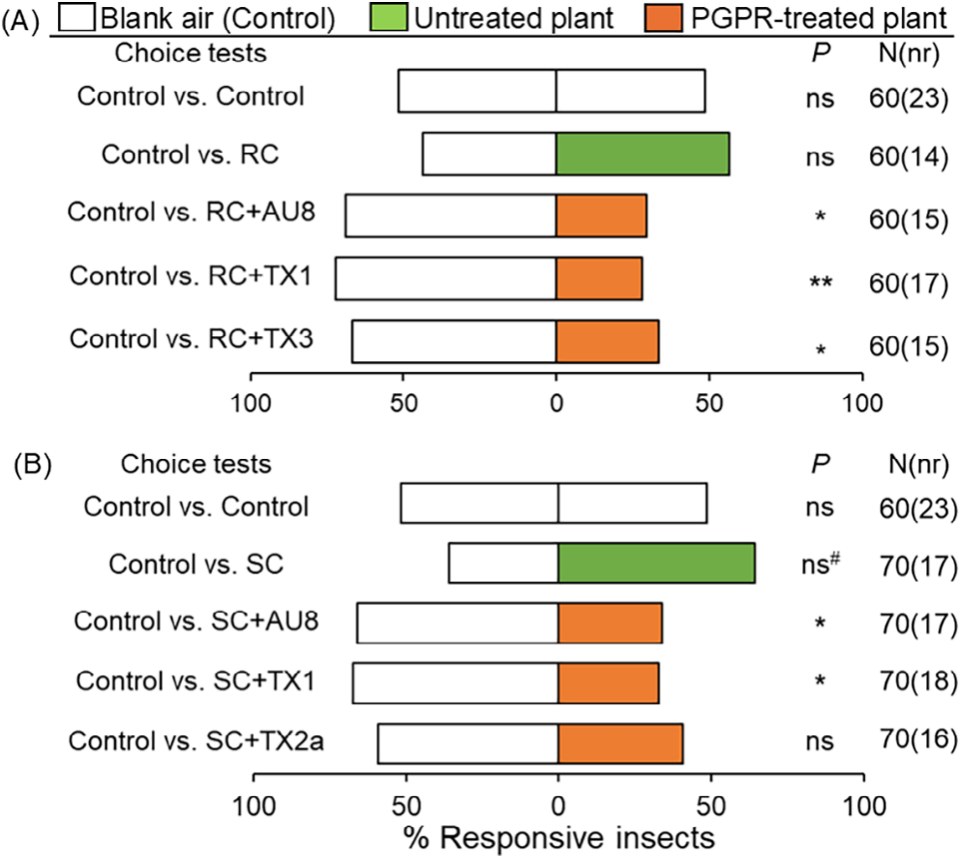
Behavioral responses (%) of *Spodoptera exigua* to volatiles of untreated and PGPR consortium‐treated plants. (A): Percentage of *S. exigua* responding to volatiles of resistant cotton (RC, cv. Acala 1517‐08) plants treated with AU8, TX1 or TX3, or left untreated compared to blank air (control). (B): Percentage of *S. exigua* responding to volatiles of susceptible cotton (SC, cv. Acala 1517–18 GLS) plants treated with AU8, TX1 or TX2a, or left untreated compared to blank air. N = total numbers of insects tested, and nr = number of non‐responsive insects (*i.e*., insects that made no‐choice). *P* stands for level of significance with ns = no significant difference (*P >* 0.05), ns^#^ = difference was almost significant (*P* = 0.054); * and ** = significant differences at *P* < 0.05 and *P* < 0.01, respectively, from χ^2^ test at α = 0.05.

*Spodopera exigua* females displayed significant avoidance behavior to volatiles of resistant plants treated with TX1, TX3 or AU8 compared to blank air (control) (χ^2^ = 6.56, *P* = 0.01; χ^2^ = 7.53, *P* = 0.006; and χ^2^ = 4.36, *P* = 0.037; respectively) (Fig. 3(A)). Similarly, significant numbers of females avoided volatiles of the susceptible plants treated with AU8 or TX1 when compared to blank air (χ^2^ = 4.83, *P* = 0.028; and χ^2^ = 5.56, *P* = 0.018; respectively), but there was no significant difference when the insect was given a choice between volatiles of TX2a‐treated plants and blank air (χ^2^ = 0.9, *P* = 0.34) (Fig. 3(B)). Volatiles of untreated susceptible plants attracted majority (~ 64%) of *S. exigua* when compared to clean air, but the difference was not significant (χ^2^ = 3.7, *P* = 0.054). However, volatiles of untreated resistant plants did not attract more *S. exigua* than the blank air (χ^2^ = 0.54, *P* = 0.461).

### 3.4 Analysis of cotton volatile organic compounds

Differences in volatile compositions were mostly quantitative between PGPR‐treated plants and untreated plants (Tables 2 and 3). A total of 13 compounds were identified, belonging to five chemical classes: aldehydes (benzaldehyde, nonanal and decanal), esters ((*Z*)‐3‐hexenyl acetate), monoterpenes (*α*‐pinene, *β*‐pinene, *β‐*ocimene, *β‐*myrcene and D‐limonene), homoterpenes (DMNT) and sesquiterpenes (*α*‐humulene, *α*‐farnesene and *β*‐caryophyllene). All 13 compounds were recorded in the volatile extracts of the resistant plants (Table 2), whereas 7 were detected in the susceptible plants (Table 3).

**Table 2:** Comparative means (±SE; *n* = 5) of volatile release rates (ng/plant/h) from resistant cotton (cv. Acala 1517‐08) plants treated with the different PGPR consortia (TX1, TX2a, TX3, AU8, AU9a) or left untreated (control)

No	RT	Compound name^†^	KI cal^§^	KI lit^¶^	Control	TX1	TX2a	TX3	AU8	AU9a
1	7.38	*α*‐Pinene^‡^	935	937	71.01 ± 12.84^c^	309.68 ± 69.61^ab^	213.03 ± 47.94^b^	1269.61 ± 382.16^a^	222.27 ± 44.52^b^	125.97 ± 19.63^bc^
2	8.16	Benzaldehyde^‡^	961	960	32.86 ± 4.66	32.28 ± 7.45	30.83 ± 12.77	281.01 ± 48.76	72.24 ± 50.76	90.95 ± 59.54
3	8.62	*β*‐Pinene^‡^	976	978	23.5 ± 5.72^b^	46.25 ± 12.55^b^	47.71 ± 17.47^b^	341.76 ± 65.27^a^	88.42 ± 45.14^b^	39.66 ± 20.14^b^
4	9.14	*β‐*Myrcene^‡^	993	992	48.02 ± 17.28	82.93 ± 27.93	58.43 ± 20.05	308.22 ± 104.4	125.66 ± 79.08	46.75 ± 20.08
5	9.66	(*Z*)‐3‐Hexenyl acetate^‡^	1011	1008	16.98 ± 3.62^b^	33.59 ± 11.74^b^	43.1 ± 25.8^b^	215.18 ± 94.07^a^	85.84 ± 49.8^ab^	45.69 ± 16.09^ab^
6	10.21	D‐Limonene^‡^	1028	1030	17.2 ± 4.7^b^	27.83 ± 7.7^b^	25.25 ± 8.81^b^	159.18 ± 33.47^a^	39.76 ± 22.81^b^	27.73 ± 9.14^b^
7	10.86	*β‐*Ocimene^‡^	1049	1044	26.5 ± 10.41	16.13 ± 4.29	17.60 ± 5.73	124.31 ± 36.56	33.08 ± 26.61	23.3 ± 10.68
8	12.55	Nonanal^‡^	1106	1105	11.24 ± 3.8^bc^	2.92 ± 0.27^d^	2.84 ± 0.68^d^	67.84 ± 23.31^ab^	7.79 ± 3.4^cd^	14.12 ± 4.64^bcd^
9	12.89	DMNT*	1118	1116	39.65 ± 16.9	46.81 ± 18.04	137.53 ± 53.49	327.53 ± 188.5	140.09 ± 71.68	34.5 ± 9.86
10	15.49	Decanal^‡^	1207	1208	12.45 ± 5.47^ab^	12.26 ± 7.79^ab^	12.9 ± 6.48^ab^	42.72 ± 8.4^a^	8.23 ± 8.23^b^	10.34 ± 3.72^ab^
11	21.2	*β‐*Caryophyllene^‡^	1420	1420	59.63 ± 19.22	70.4 ± 29.08	43.79 ± 17.24	261.74 ± 88.65	94.42 ± 59.52	56.23 ± 16.58
12	22.05	α‐Humulene^‡^	1454	1455	15.45 ± 5.17	17.56 ± 7.01	9.46 ± 3.42	11.83 ± 11.83	21.16 ± 12.80	17.36 ± 4.74
13	23.58	α‐Farnesene^‡^	1516	1515	15.31 ± 4.98	13.13 ± 5.92	10.92 ± 4.1	10.34 ± 10.34	15.59 ± 15.05	13.29 ± 4.21
Mean total amount of volatiles (ng/plant/h)					389.8 ± 82.69^b^	711.76 ± 165.46^b^	653.4 ± 202.62^b^	3421.29 ± 612.18^a^	954.56 ± 417.29^b^	545.89 ± 147.27^b^

* DMNT = (*E*)‐4,8‐Dimethylnona‐1,3,7‐triene.

^†^ Identification of compounds based on the retention time (RT), retention indices and mass spectra using library, *i.e*., NIST20, and comparison with published mass spectra and retention indices from online NIST library.

^‡^ Compounds confirmed using synthetic standards. Treatments with different lower‐case letters per volatile compound are significantly different using the non‐parametric Kruskal–Wallis one way ANOVA test at *P* ≤ 0.05.

^§^ Retention index calculated relative to C8–C20 *n*‐alkanes run on a HP‐5MS non‐polar capillary column.

^¶^ Retention index obtained from the literature.

**Table 3:** Comparative means (±SE; *n* = 5) of volatile release rates (ng/plant/h) from susceptible cotton (cv. Acala 1517‐18 GLS) plants treated with the different PGPR consortia (TX1, TX2a, TX3, AU8, AU9a) or left untreated (control).

No	RT	Compound name^†^	KI cal^§^	KI lit^¶^	Control	TX1	TX2a	TX3	AU8	AU9a
1	7.38	*α*‐Pinene^‡^	935	937	nd	nd	nd	nd	nd	nd
2	8.16	Benzaldehyde^‡^	961	960	25.74 ± 6^c^	144.44 ± 30.01^ab^	327.77 ± 29.95^a^	58.6 ± 18.35^bc^	119.93 ± 45.64^bc^	276.6 ± 96.99^a^
3	8.62	*β*‐Pinene^‡^	976	978	nd	nd	nd	nd	nd	nd
4	9.14	*β‐*Myrcene^‡^	993	992	nd	nd	nd	nd	nd	nd
5	9.66	(*Z*)‐3‐Hexenyl acetate^‡^	1011	1008	24.47 ± 5.52^c^	136.81 ± 26.15^a^	113.27 ± 16.27^ab^	50.03 ± 3.2^bc^	222.91 ± 83.73^a^	120.53 ± 28.02^a^
6	10.21	D‐Limonene^‡^	1028	1030	4.13 ± 2.08^b^	13.52 ± 3.53^ab^	11.08 ± 2.57^ab^	3.75 ± 2.27^b^	5.06 ± 2.26^b^	19.64 ± 2.16^a^
7	10.86	*β‐*Ocimene^‡^	1049	1044	7.71 ± 2.72^c^	83.49 ± 10.47^a^	194.84 ± 102.54^a^	13.53 ± 3.13^bc^	66.25 ± 13.23^ab^	80.52 ± 19.18^a^
8	12.55	Nonanal^‡^	1106	1105	11.46 ± 3.59^c^	67.62 ± 30.54^a^	38.49 ± 9.47^ab^	18.22 ± 4.86^bc^	19.16 ± 2.72^bc^	24.38 ± 4.9^ab^
9	12.89	DMNT*	1118	1116	63.61 ± 12.59^bc^	181.05 ± 17.87^ab^	210.31 ± 73.66^abc^	51.56 ± 9.97^c^	558.23 ± 126.2^a^	249.16 ± 43.74^a^
10	15.49	Decanal^‡^	1207	1208	13.52 ± 3.75	17.49 ± 2.4	25.51 ± 6.30	14.21 ± 3.43	13.66 ± 1.33	21.82 ± 2.15
11	21.2	*β‐*Caryophyllene^‡^	1420	1420	nd	nd	nd	nd	nd	nd
12	22.05	α‐Humulene^‡^	1454	1455	nd	nd	nd	nd	nd	nd
13	23.58	α‐Farnesene^‡^	1516	1515	nd	nd	nd	nd	nd	nd
Mean total amount of volatiles (ng/plant/h)					150.64 ± 14.17^c^	644.43 ± 96.4^ab^	921.27 ± 160.64^a^	209.9 ± 21.33^bc^	1005.22 ± 143.37^a^	792.66 ± 98.21^a^

* DMNT = (*E*)‐4,8‐Dimethylnona‐1,3,7‐triene.

^†^ Identification of compounds based on the retention time (RT), retention indices and mass spectra using library, *i.e*., NIST20, and comparison with published mass spectra and retention indices from online NIST library.

^‡^ Compounds confirmed using synthetic standards. Treatments with different lower‐case letters per volatile compound are significantly different using the non‐parametric Kruskal–Wallis one way ANOVA test at *P* ≤ 0.05.

^§^ Retention index calculated relative to C8–C20 *n*‐alkanes run on a HP‐5MS non‐polar capillary column.

^¶^ Retention index obtained from the literature.

nd, not detected.

**Figure 4:**
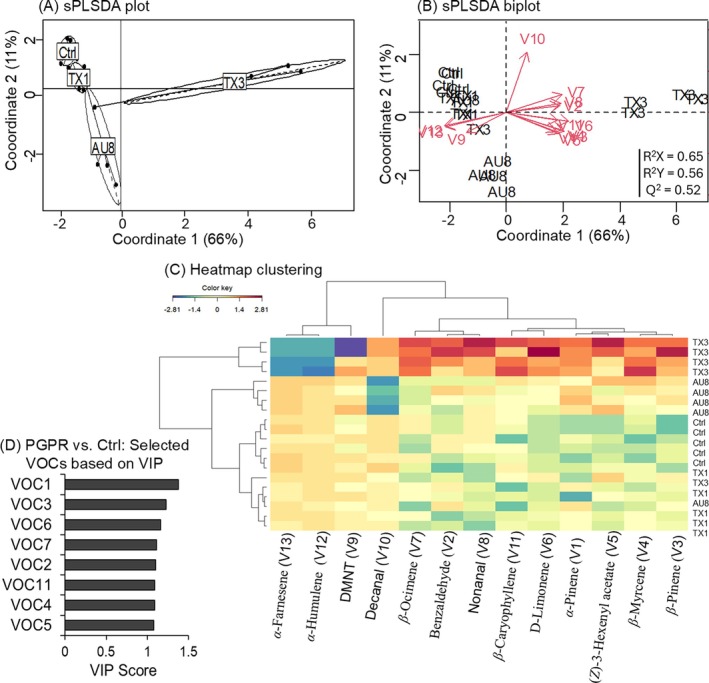
Discrimination of volatiles of the resistant cotton (RC, cv. Acala 1517–08) between plants treated with a promising PGPR consortium (AU8, TX1 or TX3) and those left untreated serving as control (Ctrl). (A) Sparse partial least square discriminant analysis (sPLS‐DA) showing the distribution of the plant treatments. (B) sPLS‐DA biplot (R^2^X = 0.65, R^2^Y = 0.56 and Q^2^ = 0.52) showing the correlation of the 13 volatile organic compounds with the plant treatments. (C) Heatmap clustering showing the abundance (in decreasing color intensity) of the 13 volatiles across replicates of untreated and PGPR‐treated plants. Indication in bracket corresponds to volatile (V) numbers as listed in Table 2. (D) Most important compounds discriminating volatile profiles of plants treated with the promising PGPR consortia from those of untreated plants, based on the variable importance in the projection (VIP >1).

Cultivar and PGPR consortium influenced overall volatile emissions. For the resistant cultivar, inoculation of plants with consortium TX3 induced a significant increase in the total amount of volatiles, unlike for inoculation with AU8, AU9a, TX1 or TX2a, relative to untreated plants (Table 2). Specifically, the monoterpenes *α*‐pinene and *β*‐pinene increased by 2‐ to 4‐fold in plants treated with TX1, TX2a or AU8, and by 15‐ to 18‐fold in plants treated with TX3. Similarly, the emission level of DMNT increased by 3‐ to 8‐fold in plants treated with TX2a, TX3 or AU8 relative to emission levels in untreated plants. An increase of 2‐ to 6‐fold in the emission level of *β‐*myrcene was recorded in AU8‐, TX1‐ or TX3‐treated plants relative to emission levels in untreated plants. The emission level of (*Z*)‐3‐hexenyl acetate increased by 2‐ to 5‐fold in plants treated with TX1, TX2a, AU8 or AU9a, and by 12‐fold in plants treated with TX3 compared to emission levels recorded from untreated plants. Regarding aldehydes and sesquiterpenes, increases in volatile emissions were found only for benzaldehyde in plants treated with AU8, AU9a or TX3 (2‐, 3‐ and 13‐fold, respectively), and for *β*‐caryophyllene in TX3‐treated plants by 4‐fold, compared to emission levels in untreated plants. The sPLS‐DA plot (R^2^X = 0.65, R^2^Y = 0.56 and Q^2^ = 0.52) separated the volatile profiles of the resistant plants into four groups: untreated plants (control), AU8‐treated plants, TX1‐treated plants and TX3‐treated plants (Fig. 4(A)), confirming that the volatile profiles of the untreated plants were distinct from those of plants treated with the promising PGPR consortia (AU8, TX1 and TX3). Furthermore, the sPLS‐DA biplot and the heatmap clustering revealed that the majority of the volatiles were associated with TX3‐treated plants, except DMNT, *α*‐humulene and *α*‐farnesene which were associated with AU8‐ and TX1‐treated plants (Fig. 4(B), (C)). The VOCs that had sPLS‐DA‐associated VIP > 1 were considered those that best distinguished the volatile profiles of untreated plants from plants treated with the promising consortia (AU8, TX1 and TX3). These included *α*‐pinene, *β*‐pinene, *β‐*myrcene, *β‐*ocimene, benzaldehyde, *β‐*caryophyllene, D‐limonene and (*Z*)‐3‐hexenyl acetate (Fig. 4(D)).

**Figure 5:**
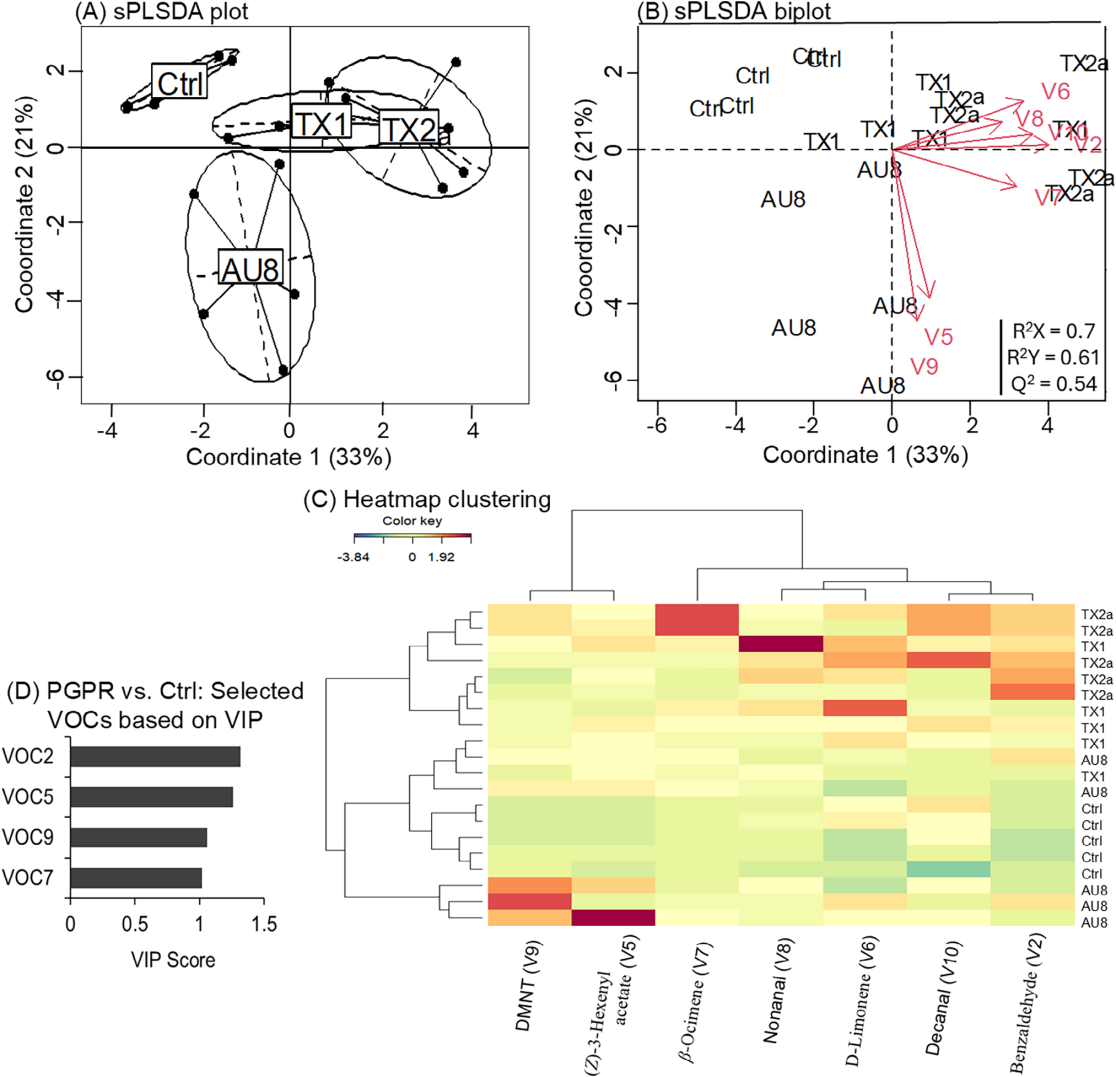
Discrimination of volatiles of the susceptible cotton (SC, cv. Acala 1517‐18 GLS) between plants treated with a promising PGPR consortium (AU8, TX1 or TX2a) and those left untreated serving as control (Ctrl). (A) Sparse partial least square discriminant analysis (sPLS‐DA) showing the distribution of the plant treatments. (B) sPLS‐DA biplot (R^2^X = 0.7, R^2^Y = 0.61 and Q^2^ = 0.54) showing the correlation of the seven volatile organic compounds with the plant treatments. (C) Heatmap clustering showing the abundance (in decreasing color intensity) of the seven volatiles across replicates of untreated and PGPR‐treated plants. Indication in bracket corresponds to volatile (V) numbers as listed in Table 3. (D) Most important compounds discriminating volatile profiles of plants treated with the promising PGPR consortia from those of untreated plants, based on the variable importance in the projection (VIP >1).

For the susceptible cultivar, there was a significant increase of 4 to 6.5‐fold in the total amount of volatiles from plants treated with the PGPR consortia relative to untreated plants, except for TX3‐treated plants for which the total volatile emission did not differ from that of the untreated plants (Table 3). Specifically, *β‐*ocimene increased by 8 to 10‐fold in plants treated with TX1 or AU8, and by 25‐fold in TX2a‐treated plants relative to emission level in untreated plants. Likewise, there was an increase of 3‐ to 9‐fold in the emission of DMNT, and 5‐ to 10‐fold in the emission of (*Z*)‐3‐hexenyl acetate in plants treated with TX1, TX2a or AU8 compared to untreated plants (Table 3). The sPLS‐DA plot (R^2^X = 0.7, R^2^Y = 0.61 and Q^2^ = 0.54) showed a clear separation of the volatile profiles of untreated plants from those of plants treated with the promising PGPR consortia (AU8, TX1 and TX2a), clustered into three groups: untreated plants (control), AU8‐treated plants, TX1‐ and TX2a‐treated plants combined (Fig. 5(A)). The sPLS‐DA biplot and the heatmap clustering further revealed that benzaldehyde, nonanal, decanal, D‐limonene and *β‐*ocimene were associated with the volatile profiles of TX1‐ or TX2a‐treated plant group, while (*Z*)‐3‐hexenyl acetate and DMNT were associated with the volatile profiles of AU8‐treated plants (Fig. 5(B), (C)). The VOCs that best discriminated between the volatile profiles of susceptible plants treated with the promising PGPR consortia (AU8, TX1 and TX2a) and those of untreated plants included *β*‐ocimene, DMNT, (Z)‐3‐hexenyl acetate, and benzaldehyde (Fig. 5(D)).

Analysis of PGPR‐induced volatiles between the susceptible and resistant plants revealed that TX3 induced the highest amount of volatiles in the resistant plants, unlike in the susceptible plants for which the total volatile emission level of TX3‐treated plants did not differ from that of untreated plants (Tables 2 and 3). Additionally, there was qualitative difference between the cultivars, whereby the monoterpenes *α*‐pinene, *β*‐pinene and *β‐*myrcene, and the sesquiterpenes *α*‐humulene, *α*‐farnesene and *β*‐caryophyllene were exclusive to the volatile profiles of the resistant cultivar, and absent in those of the susceptible cultivar (Tables 2 and 3).

## 4 DISCUSSION

Our findings revealed that crop cultivar and PGPR consortium influence the oviposition choice and olfactory responses of *S. exigua* females. We found that the PGPR consortia (*i.e*., blends of compatible strains) AU8 and TX1 significantly reduced the number of eggs laid by *S. exigua* on PGPR‐treated plants compared to untreated plants of both the susceptible and resistant cotton cultivars, demonstrating their potential broad‐spectrum efficacy in multiple cotton cultivars. The consortia AU9a and TX2a induced a reduction in egg laying only on plants of the susceptible cultivar, unlike TX3 that reduced egg laying only on plants of the resistant cultivar, compared to untreated plants. The differences in concentrations or number of strains may have contributed to the efficacy of AU8 and TX1 (four strains with total concentrations of 4 × 10^7^ CFU/mL) in both cultivars, unlike AU9a and TX2a (three strains with total concentrations of 3 × 10^7^ CFU/mL). However, TX3 also had four strains, yet was not effective in both cultivars, indicating that other factors are involved in the observed variations in efficacy. In a previous study, Akhtar *et al*.^50^ found that consortia with four PGPR strains induced comparable or higher increases of corn growth and yield compared to consortia with three and two strains, respectively. Our study contributes to the very few studies that compared the efficacy of multiple PGPR consortia of variable compositions in other crop‐insect pest systems to screen the most promising consortia for use in IPM approach.^25^, ^26^ While the effect of the PGPR consortia TX1, TX2a and TX3 on insect oviposition has not been investigated before this study, the effect of AU8 and AU9 (made of *Bacillus velezensis* AP‐295 and strains in AU9a) on insect oviposition was previously tested. For example, Nangle^51^ reported that *S. exigua* laid fewer eggs on cotton plants (cv. Max‐9) treated with AU9 compared to untreated plants, but unlike in our study, the author found no significant differences between plants treated with AU8 and untreated plants. In addition, studies on other crop‐insect pest models reported that the PGPR consortia AU8 and AU9 induced a reduction in egg laying by the European corn borer, *Ostrinia nubilalis* (Hübner) (Lepidoptera: Pyralidae), and the corn earworm, *Helicoverpa zea* Boddie (Lepidoptera: Noctuidae) on PGPR‐treated corn plants compared to untreated plants.^32^, ^52^ Aside from oviposition deterrence, we reported that the PGPR consortia impeded host plant location by *S. exigua*. We found that volatiles of the susceptible plants treated with AU8 or TX1, and resistant plants treated with AU8, TX1 or TX3 were avoided by *S. exigua* females in olfactometer bioassays. To the best of our knowledge, this study is the first to demonstrate the inhibitory effect of PGPR‐induced plant volatiles on the olfactory responses of *S. exigua*. The results are consistent with the findings reported in previous studies on other crop‐insect pest models.^14^, ^15^

The reduction in oviposition, and deterrence of olfactory responses in *S. exigua* could be explained by differences in volatile profiles between untreated and PGPR‐treated plants. PGPR‐induced headspace volatiles play a key role in the oviposition choice and host searching behavior of herbivorous insects.^14^, ^15^ Overall, we found quantitative differences in the compositions of cotton volatile emissions between untreated and PGPR‐treated plants, with emission rates of some volatile compounds being higher in plants treated with the PGPR consortia compared to emission levels recorded from untreated plants. Our findings are consistent with those previously reported by Ngumbi^53^ who found that cotton volatiles were increased in plants treated with AU8 or AU9 compared to untreated plants. However, we noted a cultivar‐dependent effect on the volatile emission. While TX3 induced the highest emissions of volatiles in the resistant cultivar, there was no difference in volatile emission levels between TX3‐treated and untreated plants in the susceptible cultivar. This contrasting effect of TX3 on volatile emission warrants further investigation. It is possible that a higher concentration of TX3 inoculum is required to induce an increase in volatile emission in the susceptible cultivar. Research on dose–response of PGPR concentrations on volatile emission has not attracted attention. However, a previous study reported a concentration‐dependent response of *Bacillus amyloliquefaciens* subsp. plantarum strain UCMB5113 on the growth of *Arabidopsis thaliana*.^54^ Qualitative differences between untreated and AU8‐ or AU9‐treated cotton plants were reported in the study of Ngumbi,^53^ whereby *β*‐pinene, (*β*)‐ocimene, *β*‐myrcene, linalool, limonene, and *α*‐humulene were not detected in untreated plants. Differences in our findings could be explained by differences in cultivars used, bacterial compositions (AU9a made of three strains in this study *vs*. AU9 made of four strains in Ngumbi), bacterial concentrations and number of booster applications (3 × 10^7^ or 4 × 10^7^ CFU/mL applied four times in this study *vs*. 1 × 10^9^ CFU/mL applied 5 or 6 times in Ngumbi). In other study models, PGPR treatment was reported to inhibit volatile emission in plants. For example, corn plants treated with AU8 or AU9 were reported to emit fewer and lower volatiles compared to untreated plants.^32^ Changes in volatile emissions are known to result from the activation of plant defense pathways such as salicylic acid (SA) pathway, jasmonic acid (JA) pathway or ethylene (ET) pathway.^55^ It is reported that the activation of JA and/or ET pathways modulates the induced systemic resistance (ISR) against insect herbivores.^56^ In cotton, the PGPR consortia AU8 and AU9 were reported to modulate IRS response through an enhanced activation of genes responsible for the biosynthesis of gossypol and JA defense related phytohormones to counter the development, oviposition and olfactory responses of herbivorous insects.^57^ In future studies, the metabolites and genes involved in the observed responses consistent with ISR‐mediated effects against *S. exigua* will be analyzed and compared between the previously reported AU8 consortium and the new consortia TX1 and TX3 tested in this study. Unlike AU8, TX1 and TX3 that consistently induced a reduction of oviposition and an inhibition of olfactory response, volatiles of TX2a‐treated plants did not elicit an avoidance behavior in *S. exigua* despite the oviposition reduction observed in no‐choice experiments. Such an inconsistent effect could be explained by the composition of TX2a‐induced volatiles. It is known that the effectiveness of a volatile blend on insect olfaction depends on the ratio and concentrations of the individual compounds mixed.^58^ The consortium TX3 exhibits pronounced cultivar‐dependent effects, inducing elevated VOC emissions and oviposition deterrence in the resistant cotton cultivar but not in the susceptible cultivar. Possible explanations include differences in root colonization efficiency or microbial compatibility to roots and levels of gene expression induced. For example, differential expressions of genes (*e.g*., DHN, LEA, MYC2 and PR1) involved in JA and SA signaling were reported between tolerant and sensitive chickpea (*Cicer arietinum*) cultivars treated with the PGPR *Pseudomonas putida*.^59^

In this study, the volatiles that best distinguished PGPR‐treated from untreated cotton plants were *α*‐pinene, *β*‐pinene, (*Z*)‐3‐hexenyl acetate, D‐limonene, *β‐*myrcene, *β‐*ocimene, benzaldehyde and *β‐*caryophyllene for the resistant cultivar, and benzaldehyde, (*Z*)‐3‐hexenyl acetate, *β*‐ocimene and DMNT for the susceptible cultivar. Among these key compounds, *α*‐pinene *β*‐pinene, limonene, benzaldehyde, *β‐*myrcene, *β*‐caryophyllene and (*Z*)‐3‐hexenyl acetate were found to elicit antennal responses in *S. exigua*.^27^, ^28^, ^29^ Volatile compounds like DMNT and (*Z*)‐3‐hexenyl acetate were found to inhibit oviposition behavior and olfactory responses in other closely related insect species like the fall armyworm, *Spodoptera frugiperda* (J. Smith) (Lepidoptera: Noctuidae) and the cotton leafworm, *Spodoptera littoralis* (Boisduval) (Lepidoptera: Noctuidae).^60^, ^61^ Other compounds reported to trigger antennal responses in *S. exigua* include nonanal, eucalyptol, *p*‐cymene, geranyl acetate, and methyl stearate.^27^, ^28^ When searching for hosts in nature, insects use odorant blends which are known to be more reliable for olfaction and to elicit faster olfactory processing responses in insects than single odorants.^62^ To the best of our knowledge, before this study, only a three‐component blend made of eucalyptol, *p*‐cymene, and geranyl acetate was reported to be repellent to *S. exigua*.^29^ Plant volatile compounds are known to play a key role in crop protection against insect herbivores.^63^, ^64^ Interestingly, the important PGPR‐induced volatile compounds identified in this study are typical herbivore‐induced plant volatiles (HIPVs) previously reported in cotton and known to serve as kairomones for natural enemies of *S. exigua*.^65^, ^66^ For example, synthetics of *α*‐pinene and *β*‐pinene applied in cotton fields were found to attract predaceous insects including the green lacewing, *Chrysoplera sinica* (Tjeder) (Neuroptera: Chrysopidae).^66^ This positive effect is favorable to the use of PGPR in an IPM package for the control of *S. exigua* in cotton.

The PGPR consortia tested in this study had limited effects on *S. exigua* larval survival rates and larval weight in both cotton cultivars. The relatively short bioassay duration (10 days) in our study may be insufficient to capture cumulative or delayed effects on development, pupation success, or adult fitness. Similarly, larval feeding damage or consumption rate, and instar developmental progression were not quantified, which constitute limitations of our study. Considering the progressive reduction in larval survival over the 10‐days experimental period, delayed effects of the PGPR consortia on *S. exigua* development cannot be excluded. Zebelo *et al*.^57^ reported a significant reduction in both the survival and weight of *S. exigua* larvae reared on cotton plants treated with AU8 or AU9 compared to those reared on untreated plants. Differences in our findings could be explained by differences in cultivars used, experimental duration (shorter in this study than in Zebelo *et al*.), bacterial compositions (AU9a made of three strains in this study *vs*. AU9 made of four strains in Zebelo *et al*.), bacterial concentrations and number of booster applications (4 × 10^7^ or 3 × 10^7^ CFU/mL applied 4 times in this study *vs*. 1 × 10^9^ CFU/mL applied six times in Zebelo *et al*.). In another crop‐insect model, Cortez *et al*.^67^ reported that inoculation of corn plants with a PGPR consortium made of *B. pumilus, B. subtilis*, and *F. solisalsi* led to a significant reduction in the weight and growth rate of the larvae of the oriental leafworm, *Spodoptera litura* (Fabricius) (Lepidoptera: Noctuidae). On the other hand, Disi *et al*.^32^ found no significant differences in the survival and weight of *O. nubilalis* larvae fed on AU8 or AU9‐treated corn plants compared to those fed on untreated plants. It is no doubt that the crop cultivar, the composition and concentration of PGPR consortium, the experimental duration and the herbivore species affect the efficacy of PGPR on larval development. Some mechanisms deployed by PGPR to impair the weight and survival of herbivore larvae on plants include the induction of metabolites and enzymes that possess larvicidal properties.^57^, ^68^ For example, in cotton, PGPR are reported to induce an increase in the production of gossypol,^57^ a biochemical compound known to exert detrimental effect on the larval survival and weight of lepidopteran insects like *S. exigua* and the cotton bollworm, *Helicoverpa armigera* (Hübner) (Lepidoptera: Noctuidae).^69^, ^70^

## 5 CONCLUSION

In summary, this study identifies two promising PGPR consortia (AU8 and TX1) which induced a significant reduction in oviposition and an inhibitory effect on the olfactory responses of *S. exigua* in both the susceptible and resistant cotton cultivars, indicating their potential for broad‐spectrum efficacy in controlling *S. exigua* in multiple cotton cultivars. Both consortia have different bacterial species compositions dominated by *Bacillus* taxonomic group: AU8 contains *Bacillus velezensis* AP‐218, *B. velezensis* AP‐188, *B. mojavensis* AP‐209 and *Fictibacillus solisalsi* AP‐217, while TX1 contains *B. subtilis* TC04, *B. albus* TC09, *B. halotolerans* TC13 and *Paenibacillus alvei* TC44. The most discriminant volatile compounds between untreated plants and plants treated with the promising PGPR consortia were *α*‐pinene, *β*‐pinene, D‐limonene, benzaldehyde, (*Z*)‐3‐hexenyl acetate, *β*‐ocimene and DMNT, identified as potential repellent compounds for the protection of cotton cultivars against *S. exigua*. The role of these promising PGPR consortia and volatile compounds as part of an IPM approach will be further elucidated in future studies.

## CONFLICT OF INTEREST

All authors agree there are no competing interests.

## AUTHOR CONTRIBUTIONS

Conceptualization: HYF and PMA; Methodology: PMA, HYF, CX and BDK. Data collection: PMA and BDK; Data analysis: PMA; Writing of original draft: PMA; Review and editing: PMA, HYF, CX and BDK. Funding acquisition: HYF. All authors provided intellectual inputs and approved the manuscript for submission.

## Data Availability

The data that support the findings of this study are available from the corresponding author upon reasonable request.
